# Metformin Treatment Inhibits Motility and Invasion of Glioblastoma Cancer Cells

**DOI:** 10.1155/2018/5917470

**Published:** 2018-06-26

**Authors:** Marwa Al Hassan, Isabelle Fakhoury, Zeinab El Masri, Noura Ghazale, Rayane Dennaoui, Oula El Atat, Amjad Kanaan, Mirvat El-Sibai

**Affiliations:** ^1^Department of Natural Sciences, School of Arts and Sciences, Lebanese American University, Beirut, Lebanon; ^2^Department of Biomedical Sciences, Faculty of Medicine and Medical Sciences, University of Balamand, El-Kurah, Lebanon

## Abstract

Glioblastoma multiforme (GBM) is one of the most common and deadliest cancers of the central nervous system (CNS). GBMs high ability to infiltrate healthy brain tissues makes it difficult to remove surgically and account for its fatal outcomes. To improve the chances of survival, it is critical to screen for GBM-targeted anticancer agents with anti-invasive and antimigratory potential. Metformin, a commonly used drug for the treatment of diabetes, has recently emerged as a promising anticancer molecule. This prompted us, to investigate the anticancer potential of metformin against GBMs, specifically its effects on cell motility and invasion. The results show a significant decrease in the survival of SF268 cancer cells in response to treatment with metformin. Furthermore, metformin's efficiency in inhibiting 2D cell motility and cell invasion in addition to increasing cellular adhesion was also demonstrated in SF268 and U87 cells. Finally, AKT inactivation by downregulation of the phosphorylation level upon metformin treatment was also evidenced. In conclusion, this study provides insights into the anti-invasive antimetastatic potential of metformin as well as its underlying mechanism of action.

## 1. Introduction

Gliomas are brain tumors that originate within the central nervous system (CNS). Glioblastomas (GBMs), which account for about 80% of malignant gliomas, contain self-renewing cancer stem cells (CSCs) that contribute to tumor initiation and resistance to treatment [1, 2]. Death due to malignant gliomas is the third most common cause of cancer death [3, 4]. The management of malignant gliomas, especially GBMs, remains challenging despite medical and scientific advancements in cancer therapeutics. This is largely attributed to their increased resistance to chemotherapy as well as their highly invasive behavior which makes them difficult to surgically remove [5, 6]. Such shortcomings have called forth for the screening for new GBM-targeted anticancer agents with antimigratory and anti-invasive potential.

Metformin, (N, N-dimethylbiguanide) is an antihyperglycemic agent that belongs to the biguanide class. It is commonly used to treat type 2 diabetes mellitus [7, 8]. Metformin decreases hyperglycemia by suppressing glucose production in the liver, increasing insulin sensitivity and glucose uptake by the peripheral tissues, and inhibiting glucose absorption by the gastrointestinal tract as well as inhibiting the mitochondrial respiration [7, 9–11]. The drug's mechanism of action has been shown to be both adenosine monophosphate protein kinase- (AMPK) dependent and AMPK-independent [7, 10, 12]. Cancer cells resort to an increased glucose metabolism to meet their energy requirements needed for rapid expansion and proliferation [13, 14]. Consequently, metformin has emerged as a promising anticancer agent in various cancers including GBMs [15–23]. Specifically, metformin has been shown to inhibit GBMs growth *in vitro* and *in vivo* alone or in combination with other chemotherapeutics as well as radiation therapy [24–31]. Furthermore, metformin's anticancer potential has also been demonstrated against glioma cancer stem cells and brain tumor-initiating cells [26, 27, 30, 32–35]. However, the effects of metformin on glioma cell motility and invasion as well as its mechanism of action remain poorly understood.

Glioma invasion is a multistep process regulated by extracellular and intracellular interactions [36–38]. It starts with the detachment of cancer cells from primary tumor sites, their binding to the extracellular matrix (ECM) and subsequent degradation of the ECM to finalize the invasion process. Cell motility is essential for the migration and invasion of cancer cells. Cell motility requires the formation and liberation of cell protrusions from adhesion structures [36, 37, 39, 40].

In this study, we sought to assess the anticancer potential of metformin on SF268 brain cancer cells and investigate the drug's antimigratory and anti-invasive potential as well as its mechanism of action. To this aim, we first evaluated metformin's cytotoxic effects against SF268 cancer cells using WST-1 proliferation assay. We then performed 2D motility, adhesion, and invasion assays to determine the drug's antimigratory and anti-invasive potential. Finally, we examined the mechanism of action of metformin, by assessing its effects on the PI3K pathway, one of the most deregulated signaling pathways in glioblastoma. Specifically, we studied the involvement of the antiapoptotic protein AKT of the PI3K pathway in metformin's anticancer, anti-invasive, and antimigratory potential.

## 2. Materials and Methods

### 2.1. Cell Culture

Human astrocytoma cell lines SF268 and U87 were purchased from the American Type Culture Collection (Manassas, VA, USA). The cells were cultured in DMEM (Dulbecco's Modified Eagle's Medium) supplemented with 10% FBS and 100 U penicillin/streptomycin and were maintained under standard cell culture conditions at 37°C and 5% CO_2_ in a humid environment.

### 2.2. Antibodies and Reagents

Rabbit monoclonal antibody against pan-Akt and rabbit monoclonal antibody against Akt1 phosphorylated at S473 were purchased from Abcam (Cambridge, UK). Anti-rabbit HRP-conjugated secondary antibody was obtained from Promega (Promega, CO., WI, USA). Collagen was purchased from Invitrogen (Rockville, MD, USA), metformin (1, 1-dimethylbiguanide hydrochloride) (purity > 99%) from Sigma-Aldrich (St. Louis, MO, USA), WST-1 from Roche (Germany), the ECL chemiluminescent reagent from GE Healthcare (Little Chalfont, UK), X-ray films from Agfa HealthCare (Mortsel, Belgium), and the collagen-based invasion assay from Millipore (Burlington, MA, USA).

### 2.3. Proliferation Assay

Cells were seeded in flat-bottom 96-well plates (growth area: 0.6 cm^2^) at a density of 10^5^cells/ml before treatment with or without metformin dissolved in DMSO. Following treatment period, 10 *μ*l of Cell Proliferation Reagent (WST-1) was added to each well. The plates were then incubated for 2 h at 37°C and 5% CO_2_, and absorbance was read at 450 nm using Multiskan FC microplate ELISA reader from Thermo Fisher Scientific (Rockford, IL, USA). Results were normalized to the corresponding controls, and the percent of cell proliferation was reported. For the next set of experiments, we followed the methods of Khoury et al. [41].

### 2.4. Western Blot

Control and treated cells were scraped and lysed in buffer consisting of 4% SDS, 10% *β*-mercaptoethanol, 20% glycerol, 0.004% bromophenol blue, and 0.125 M Tris-HCl at a pH of 6.8. Cell lysates were boiled for 5 min before separating protein samples by SDS-PAGE on 8% or 15% denaturing polyacrylamide gels. Proteins were then transferred to PVDF membranes overnight at 30 V before blocking with 5% nonfat dry milk in PBS containing 0.1% Tween-20 for 1 h at room temperature. Following, the membranes were incubated with primary antibody at a concentration of 1 : 500 for 2 h at room temperature before washing and incubation with the appropriate secondary antibody at a concentration of 1 : 1000 for 2 h. Finally, the membranes were washed, and the bands were visualized by treatment with Western blotting ECL chemiluminescent reagent from GE Healthcare (Little Chalfont, UK). The results were obtained on X-ray films from Agfa HealthCare (Mortsel, Belgium). Protein expression levels were quantified by densitometry analysis using ImageJ.

### 2.5. Wound Healing Assay

Cells were grown to confluency on culture plates, and a wound was made in the monolayer with a sterile pipette tip. The cells were then washed twice with PBS to remove debris and supplemented with new medium. Phase-contrast images of the wounded area were taken at 0 and 24 h after wounding. ImageJ was used to quantify wound widths at 12 different points for each wound, and the average rate of wound closure was calculated in *μ*m/h.

### 2.6. Random Motility Assay

For motility analysis, cells were treated with wortmannin or metformin or left untreated. Images of cells moving randomly in serum were collected every 60 seconds or every 3 minutes for 2 h using a 20x objective. During imaging, the temperature was controlled using a heating stage which was set at 37°C. The medium was buffered using HEPES and overlaid with mineral oil. Cell movement was quantified using the ROI tracker plugin in the ImageJ software, written by Dr. David Entenberg. This was used to calculate the total distance travelled by individual cells. The net distance travelled by the cell was calculated by measuring the distance travelled between the first and the last frames.

### 2.7. Invasion Assay

Invasion assay was performed 48 h after treatment with metformin using the collagen-based invasion assay kit from Millipore (Burlington, MA, USA) according to the manufacturer's protocol. Briefly, SF268 cells were starved in serum-free medium for 24 h before harvesting and resuspension in quenching medium (serum-free). Culture plate inserts were rehydrated using 300 *μ*l of serum-free medium for 30 min at room temperature before plating the cells at a density of 0.6 × 10^6^ cells/ml. Specifically, 250 *μ*l of the serum-free medium was removed from inserts and replaced by 250 *μ*l of cell suspension. Inserts were then placed in a 24-well plate containing 500 *μ*l of complete medium in each well before incubation for 24 h at 37°C in a CO_2_ incubator. Following incubation, nonmigrating cells inside the upper cup were removed using a cotton swab and cells migrating through the membrane to the bottom surface of the cup were stained for 20 min at room temperature with 400 *μ*l of cell stain provided with the kit. The stain was then extracted with extraction buffer and 100 *μ*l of extracted stain was transferred to a 96-well plate suitable for colorimetric measurement using the Multiskan FC microplate ELISA reader, and optical density was measured at 560 nm.

### 2.8. Adhesion Assay

To perform the adhesion assay, 96-well plates were covered with collagen using Collagen Solution Type I from rat tail and incubated overnight at 37°C. After washing with washing buffer (0.1% BSA in DMEM), the plates were blocked with 0.5% BSA in DMEM at 37°C in a CO_2_ incubator for 1 h. Next, the plates were washed and chilled on ice. In parallel, SF268 cells were trypsinized and diluted to the density of 4 × 10^5^cell/ml before adding 50 *μ*l of the cell suspension to each well and incubating at 37°C in a 5% CO_2_ incubator for 30 min. Plates were then shaken and washed 3 times. Next, the cells were fixed with 4% paraformaldehyde at room temperature for 10 min, washed, and stained with crystal violet (5 mg/ml in 2% ethanol) for 10 min. Following staining, the plates were washed with water and left to dry. Finally, crystal violet was solubilized by incubating the cells with 2% SDS for 30 min. The absorption of the plates was read at 550 nm using the Multiskan FC microplate ELISA reader.

### 2.9. Statistical Analysis

All the results reported represent the average values of three independent experiments. All error estimates are given as mean ± standard error of the mean (SEM). Statistical analysis was performed using the *t*-test or the one-way analysis of variance (ANOVA). Results showed statistical significance with a *p* value ≤ 0.05.

## 3. Results

### 3.1. Metformin Treatment Decreases Cell Viability in SF268 and U87 Cells

**Figure 1:**
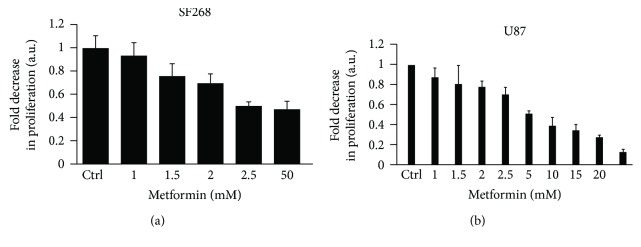
Metformin treatment decreases cell viability in SF268 and U87 cells. Cells were treated with increasing concentrations of metformin (1, 1.5, 2, 2.5, 5, 10, 15, 20, or 50 mM) for 24 h or left untreated. Cell proliferation was assessed using the WST-1 reagent. The data represents the mean ± SEM from 3 independent experiments.

First, we investigated the anticancer potential of metformin against human glioblastoma SF268 and U87 cancer cells. To this aim, cells were treated with increasing concentrations of metformin (1, 1.5, 2, 2.5, 5, 10, 15, 20, or 50 mM) for 24 h before assessing cell proliferation and viability. The results presented in Figure 1(a) for SF268 and 1B for U87 show that metformin significantly reduces cell viability of both cell lines in a dose-dependent manner as compared to the untreated control. In SF268, metformin exerted a maximum cytotoxic effect at a concentration of 2.5 mM, whereby the proliferation of glioblastoma cells decreases about twofold in response to treatment with metformin as compared to the untreated control. The effect on cytotoxicity in SF268 plateaued beyond the 2.5 mM concentration. This concentration was thus chosen for further investigation in the study.

### 3.2. Metformin Treatment Inhibits Cell Motility in SF268 and U87 Cells

**Figure 2:**
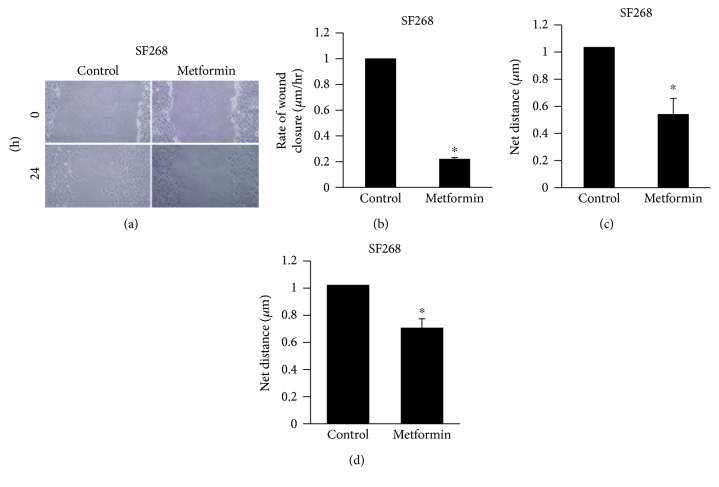
Metformin treatment inhibits cell motility in SF268 and U87 cells. (a) Control and metformin-treated SF268 cells were allowed to form monolayers before wounding with a micropipette. The same frame was imaged directly (upper micrographs) or 24 h (lower micrographs) after wound. (b) Mobility in (a) was quantified by measuring the width of single wound at 12 distinct points. The average rate of wound closure was calculated in *μ*m/hr. Data are the mean ± SEM from 3 different wound healing experiments. ^∗^ indicates that the values are significant with *p* < 0.05. (c) SF268 cells were treated with metformin (2.5 mM) (Supplemental movie S2) for 24 h or left untreated (Supplemental movie S1). Quantitation of their net path is expressed in *μ*m. ^∗^ indicates that the values are significant with *p* < 0.001. U87 cells were treated with metformin (2.5 mM) (Supplemental movie S4) for 24 h or left untreated (Supplemental movie S3). Quantitation of their net path is expressed in *μ*m. ^∗^ indicates that the values are significant with *p* < 0.001.

After determining the cytotoxic potential of metformin against SF268 glioblastoma cancer cell line, we tested the drug's ability to modulate cell motility. Therefore, SF268 cancer cells were exposed to metformin and the motility of treated versus untreated cancer cells was evaluated in 2D using wound healing and time-lapse assays. Figures 2(a)–2(c) (as well as supplemental movies S1 and S2) show that exposure of SF268 glioblastoma cells to 2.5 mM metformin for 24 h significantly inhibits cell motility. Quantitatively, the rates of wound closure reached 1 *μ*m/h and 0.22 *μ*m/h for control and metformin-treated cells, respectively. Also, the total migrated distance decreased by approximately 50% upon treatment with metformin. The time-lapse analysis traces individual cell migration, thus eliminating the potential interference from the effect on proliferation. We also wanted to see the effect of metformin on cellular migration of U87 cells. The time-lapse assay showed a 40% decrease in the total migrated distance of U87 cells after treatment with metformin (2.5 mM) for 24 h (Figure 2(d) and supplemental movies S3 and S4).

### 3.3. Metformin Treatment Decreases Cellular Invasion in SF268 and U87 Cells

**Figure 3:**
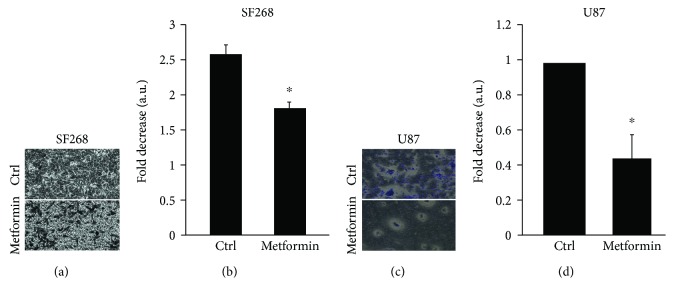
Metformin treatment decreases cellular invasion in SF268 and U87 cells. Representative images showing invasive cells at the bottom side of the membrane for SF268 cells in (a) and U87 cells in (c). The cells were stained with crystal violet as per the manufacturer's recommendations (b) and (d) show quantitation of stained SF268 and U87 cells, respectively, by colorimetric measurement using ELISA (560 nm). Data is measured in arbitrary units and normalized to the control. Data are the mean ± SEM from 3 independent experiments. ^∗^ indicates that the values are significant with *p* < 0.001.

Having established that metformin inhibits cell motility in 2D, we further studied the effect of metformin on invasion, one of the main cancer hallmarks. Using a transwell migration assay and FBS as a chemoattractant in the lower wells, SF268 and U87 cells were treated with 2.5 mM metformin for 24 h before assessing their ability to invade *in vitro* in a collagen-based invasion assay. Figures 3 and 3 show less invading cells upon treatment with metformin as compared to the control.. Quantitatively, metformin inhibits cell invasion by around 30% in SF268 as compared to the untreated control and by 50% in U87 cells (Figures 3(b) and 3(d), resp.).

### 3.4. Metformin Treatment Increases SF268 Adhesion to Collagen

**Figure 4:**
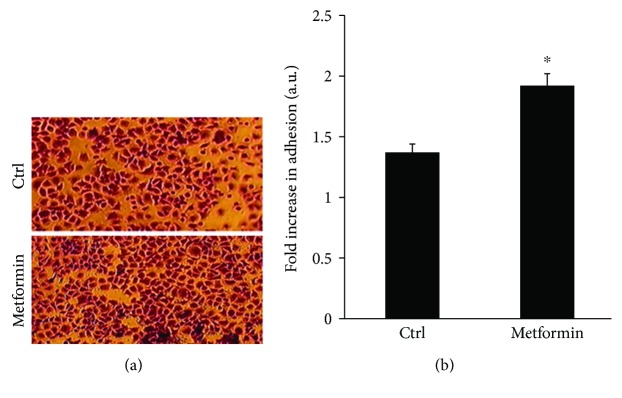
Metformin treatment increases SF268 adhesion to collagen. (a) Representative images of cells that were fixed and stained with crystal violet to detect adhesion. (b) Quantitation of stained cells by colorimetric measurement using ELISA (560 nm). Data is measured in arbitrary units and normalized to the control. Data are the mean ± SEM from 3 independent experiments. The results were significant with *p* = 0.02.

To further investigate the inhibition of 2D cell motility and invasion in response to treatment with metformin, we assessed metformin's effects on the adhesion of SF268 cells to collagen, a major component of the ECM. Results in Figure 4(a) show an increase in the stabilization and adhesion of SF268 metformin-treated cells (2.5 mM for 24 h) to collagen as compared to the control. As shown in Figure 4(b), about 35% increase in adhesion was noted following treatment with metformin as compared to the untreated control.

### 3.5. Effect of Metformin on SF268 Cell Motility Is Mimicked by Inhibiting PI3K

**Figure 5:**
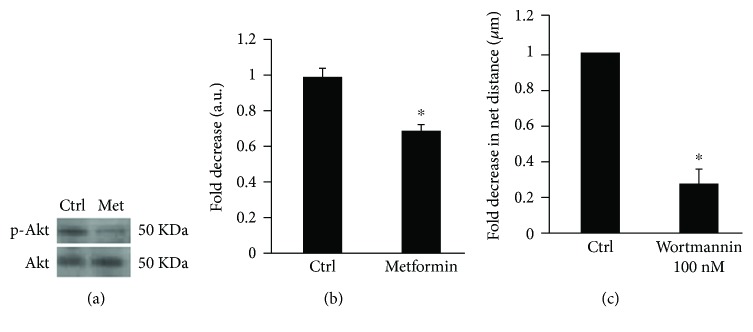
Effect of metformin on SF268 cell motility is mimicked by inhibiting PI3K. (a) Cells were treated with metformin for 24 h before blotting for Akt and p-Akt. (b) Densitometry analysis was performed using the ImageJ software, and the values were normalized to the control. Data are the mean ± SEM from 3 independent experiments. The results were significant with *p* = 0.03. (c) SF268 cells were treated with wortmannin (100 nM) (Supplemental movie S6) or with DMSO alone (Supplemental movie S5). Quantitation of their net path is expressed in *μ*m. Data are the mean ± SEM. ^∗^ indicates that the values are significant with *p* < 0.001.

Since previous work showed that metformin inhibits cellular migration and invasion by inhibiting Akt, we wanted to see if this model applies to SF268 glioblastoma cells. We examined the effect of metformin on the PI3K/Akt signaling pathway.  Figure 5(a) shows that treatment of SF268 cells with 2.5 mM of metformin for 24 h has no effect on the expression levels of the Akt protein. However, as seen in both Figures 5(a) and 5(b), treatment with metformin significantly inhibits the phosphorylation of Akt which reflects the activation of the PI3K pathway. Specifically, Akt phosphorylation was reduced by 30% upon treatment with metformin indicating a partial inactivation of this protein.

To assess the role of the PI3K/Akt pathway in glioblastoma migration, we inhibited PI3K using wortmannin. The inhibition leads to a significant decrease in cellular motility in a 2D random motility assay as seen in Figure 5(c) and Supplemental movies S5 and S6. Our results indicate that PI3K/Akt pathway plays an important role in GBM invasiveness.

## 4. Discussion

This work provides an understanding of metformin treatment effects on SF268 and U87 glioblastoma cancer cell viability, motility and invasion. It also establishes the Akt antiapoptotic protein of the PI3K signaling pathway as a key mediator of metformin's mechanism of action. To our knowledge, this is one of the few studies investigating the anticancer potential of metformin against the aggressive SF268 and U87 and assessing the anti-invasive and antimigratory effects of this drug in this GBM cancer model.

First, we examined the anticancer potential of the drug *in vitro* and established that metformin is cytotoxic to SF268 and U87 cancer cells as evidenced by the reduced cell viability following treatment with metformin. This was consistent with a similar study which shows that treatment of T98G glioblastoma multiform cells with metformin decreases cell viability and triggers apoptotic morphological alterations in the cells [28]. The decrease in SF268 and U87 cell viability following treatment with 2.5 mM was also similar to the results obtained by another group which have reported a decrease in U87MG, T98G, and U251 cancer cell viability by 46%, 92% and 99%, respectively, upon treatment with 2.5 mM metformin as compared to the control [42].

Our findings further revealed that metformin decreases 2D cell motility by 80%, hence almost abrogating it. This property has been previously reported in other cancer models including pancreatic, breast, renal cell, colon, lung, ovarian, glioma, and prostate [43–47]. For instance, metformin was shown to inhibit wound healing in cholangiocarcinoma cells and metformin in combination with cisplatin was shown to inhibit the migration of nasopharyngeal carcinoma cells [46, 47]. This is thus the first evidence of metformin's antimigratory effects in GBMs in 2D, *in vitro*.

In addition, we observed an increase in the adhesion of SF268 cancer cells to collagen upon treatment with metformin. Focal adhesion dissolution is required for cell movement; hence, the increase in adhesion is in line with the reduced cell motility findings discussed earlier. However, one other group found that cell adhesion and invasion of U251 GBM cancer cells were suppressed following treatment with metformin [48]. The increase in adhesion we report herein is consistent with previous work performed in our lab which shows that the RhoGAP STARD13 maintains RhoA active and prevents focal adhesion dissolution [49, 50]. Indeed, the metformin-treated cells exhibited a more elongated phenotype (Supplemental movie S2) which is reminiscent of the StarD13 KnDn phenotype in SF268 cells previously observed in our laboratory, which can be explained by the increase in adhesion and lack of detachment at the tail while the cells migrate. We are thus currently testing the effect of metformin on STARD13 and RhoA and the interplay with cell adhesion and cell motility.

In parallel, we investigated the effects of metformin on the 3D motility or the invasion of SF268 and U87 cancer cells and demonstrated the efficient reduction cell invasion in response to treatment with metformin. This was consistent with the literature and was reported in melanoma, ovarian cancer, U251 brain cancer, and others [48, 51, 52].

Finally, we showed that metformin inactivates AKT, a major signaling molecule of the PI3K pathway suggesting a potential role for PI3K inhibition in the mediation of the anticancer potential and anti-invasive as well as antimigratory effects exerted by metformin. The importance of the PI3K/Akt pathway in glioblastoma was validated when we treated cells with wortmannin, a PI3K inhibitor. Wortmannin treatment decreased cellular motility and inhibited EGF stimulated protrusions correlating with our metformin results. Similar studies have supported this conclusion whereby the effects of metformin correlated with a significant inhibition of Akt-dependent cell survival pathway [34].

## 5. Conclusions

This study elucidates the anticancer potential of metformin treatment in a new GBMs *in vitro* model which have not been previously studied. It also demonstrates the drug's anti-invasive and antimigratory potentials. Invasion is a major obstacle for GBM therapy; hence, these findings enhance metformin's chances as a therapeutic candidate for GBM treatment. Further studies are thus needed to investigate metformin's efficiency in treating GBMs *in vivo*.

## Data Availability

The data that support the findings of this study are available from the corresponding author upon reasonable request.

## Supplementary Materials

Supplementary 1 Supplemental movie S1. Control SF268 cells undergoing 2D motility.

Supplementary 2 Supplemental movie S2. SF268 cells treated with Metformin undergoing 2D motility.

Supplementary 3 Supplemental movie S3. U87 cells undergoing 2D motility.

Supplementary 4 Supplemental movie S4. U87 cells treated with Metformin undergoing 2D motility.

Supplementary 5 Supplemental movie S5. SF268 cells treated with DMSO undergoing 2D motility.

Supplementary 6 Supplemental movie S6. SF268 cells treated with wortmannin undergoing 2D motility.

## References

[1] Abou-Antoun T. J., Hale J. S., Lathia J. D., Dombrowski S. M. (2017). Brain cancer stem cells in adults and children: cell biology and therapeutic implications. *Neurotherapeutics*.

[2] Lathia J. D., Mack S. C., Mulkearns-Hubert E. E., Valentim C. L. L., Rich J. N. (2015). Cancer stem cells in glioblastoma. *Genes & Development*.

[3] Ostrom Q. T., Gittleman H., de Blank P. M. (2015). American brain tumor association adolescent and young adult primary brain and central nervous system tumors diagnosed in the United States in 2008-2012. *Neuro-Oncology*.

[4] Ostrom Q. T., Gittleman H., Xu J. (2016). CBTRUS statistical report: primary brain and other central nervous system tumors diagnosed in the United States in 2009-2013. *Neuro-Oncology*.

[5] Angelopoulou E., Piperi C. (2018). Emerging role of plexins signaling in glioma progression and therapy. *Cancer Letters*.

[6] Omuro A., DeAngelis L. M. (2013). Glioblastoma and other malignant gliomas: a clinical review. *JAMA*.

[7] Rojas L. B. A., Gomes M. B. (2013). Metformin: an old but still the best treatment for type 2 diabetes. *Diabetology & Metabolic Syndrome*.

[8] Sanchez-Rangel E., Inzucchi S. E. (2017). Metformin: clinical use in type 2 diabetes. *Diabetologia*.

[9] Wróbel M. P., Marek B., Kajdaniuk D., Rokicka D., Szymborska-Kajanek A., Strojek K. (2017). Metformin - a new old drug. *Endokrynologia Polska*.

[10] Rena G., Hardie D. G., Pearson E. R. (2017). The mechanisms of action of metformin. *Diabetologia*.

[11] El-Mir M.-Y., Nogueira V., Fontaine E., Avéret N., Rigoulet M., Leverve X. (2000). Dimethylbiguanide inhibits cell respiration via an indirect effect targeted on the respiratory chain complex I. *The Journal of Biological Chemistry*.

[12] Foretz M., Hébrard S., Leclerc J. (2010). Metformin inhibits hepatic gluconeogenesis in mice independently of the LKB1/AMPK pathway via a decrease in hepatic energy state. *The Journal of Clinical Investigation*.

[13] Abbadi S., Rodarte J. J., Abutaleb A. (2014). Glucose-6-phosphatase is a key metabolic regulator of glioblastoma invasion. *Molecular Cancer Research*.

[14] Hanahan D., Weinberg R. A. (2011). Hallmarks of cancer: the next generation. *Cell*.

[15] Ge R., Wang Z., Wu S. (2015). Metformin represses cancer cells via alternate pathways in N-cadherin expressing vs. N-cadherin deficient cells. *Oncotarget*.

[16] Gou S., Cui P., Li X., Shi P., Liu T., Wang C. (2013). Low concentrations of metformin selectively inhibit CD133(+) cell proliferation in pancreatic cancer and have anticancer action. *PLoS One*.

[17] Kim T. H., Suh D. H., Kim M. K., Song Y. S. (2014). Metformin against cancer stem cells through the modulation of energy metabolism: special considerations on ovarian cancer. *BioMed Research International*.

[18] Kong F., Gao F., Liu H. (2015). Metformin use improves the survival of diabetic combined small-cell lung cancer patients. *Tumour Biology*.

[19] Leone A., di Gennaro E., Bruzzese F., Avallone A., Budillon A. (2014). New perspective for an old antidiabetic drug: metformin as anticancer agent. *Cancer Treatment and Research*.

[20] Miranda V. C., Barroso-Sousa R., Glasberg J., Riechelmann R. P. (2014). Exploring the role of metformin in anticancer treatments: a systematic review. *Drugs of Today*.

[21] Nangia-Makker P., Yu Y., Vasudevan A. (2014). Metformin: a potential therapeutic agent for recurrent colon cancer. *PLoS One*.

[22] Thompson A. M. (2014). Molecular pathways: preclinical models and clinical trials with metformin in breast cancer. *Clinical Cancer Research*.

[23] Vlotides G., Tanyeri A., Spampatti M. (2014). Anticancer effects of metformin on neuroendocrine tumor cells in vitro. *Hormones*.

[24] Ferla R., Haspinger E., Surmacz E. (2012). Metformin inhibits leptin-induced growth and migration of glioblastoma cells. *Oncology Letters*.

[25] Sesen J., Dahan P., Scotland S. J. (2015). Metformin inhibits growth of human glioblastoma cells and enhances therapeutic response. *PLoS One*.

[26] Mouhieddine T. H., Nokkari A., Itani M. M. (2015). Metformin and ara-a effectively suppress brain Cancer by targeting Cancer stem/progenitor cells. *Frontiers in Neuroscience*.

[27] Seliger C., Meyer A. L., Renner K. (2016). Metformin inhibits proliferation and migration of glioblastoma cells independently of TGF-*β*2. *Cell Cycle*.

[28] Ucbek A., Özünal Z. G., Uzun Ö., Gepdiremen A. (2014). Effect of metformin on the human T98G glioblastoma multiforme cell line. *Experimental and Therapeutic Medicine*.

[29] Yang S. H., Li S., Lu G. (2016). Metformin treatment reduces temozolomide resistance of glioblastoma cells. *Oncotarget*.

[30] Yu Z., Zhao G., Xie G. (2015). Metformin and temozolomide act synergistically to inhibit growth of glioma cells and glioma stem cells in vitro and in vivo. *Oncotarget*.

[31] Adeberg S., Bernhardt D., Harrabi S. B. (2017). Metformin enhanced in vitro radiosensitivity associates with G2/M cell cycle arrest and elevated Adenosine-5′-monophosphate-activated protein kinase levels in glioblastoma. *Radiology and Oncology*.

[32] Carmignani M., Volpe A. R., Aldea M. (2014). Glioblastoma stem cells: a new target for metformin and arsenic trioxide. *Journal of Biological Regulators and Homeostatic Agents*.

[33] Sato A., Sunayama J., Okada M. (2012). Glioma-initiating cell elimination by metformin activation of FOXO3 via AMPK. *Stem Cells Translational Medicine*.

[34] Würth R., Pattarozzi A., Gatti M. (2012). Metformin selectively affects human glioblastoma tumor-initiating cell viability: a role for metformin-induced inhibition of Akt. *Cell Cycle*.

[35] Yu Z., Zhao G., Li P. (2016). Temozolomide in combination with metformin act synergistically to inhibit proliferation and expansion of glioma stem-like cells. *Oncology Letters*.

[36] Kutys M. L., Doyle A. D., Yamada K. M. (2013). Regulation of cell adhesion and migration by cell-derived matrices. *Experimental Cell Research*.

[37] Armento A., Ehlers J., Schötterl S., Naumann U., Vleeschouwer S. (2017). Molecular Mechanisms of Glioma Cell Motility. *Glioblastoma*.

[38] Saykali B. A., El-Sibai M. (2014). Invadopodia, regulation, and assembly in cancer cell invasion. *Cell Communication & Adhesion*.

[39] Block M., Badowski C., Millonfremillon A. (2008). Podosome-type adhesions and focal adhesions, so alike yet so different. *European Journal of Cell Biology*.

[40] Hanna S., El-Sibai M. (2013). Signaling networks of Rho GTPases in cell motility. *Cellular Signalling*.

[41] Khoury N., El-Hayek S., Tarras O., El-Sabban M., El-Sibai M., Rizk S. (2014). Kefir exhibits antiproliferative and proapoptotic effects on colon adenocarcinoma cells with no significant effects on cell migration and invasion. *International Journal of Oncology*.

[42] Kennedy C. R., Tilkens S. B., Guan H., Garner J. A., Or P. M. Y., Chan A. M. (2013). Differential sensitivities of glioblastoma cell lines towards metabolic and signaling pathway inhibitions. *Cancer Letters*.

[43] Isakovic A., Harhaji L., Stevanovic D. (2007). Dual antiglioma action of metformin: cell cycle arrest and mitochondria-dependent apoptosis. *Cellular and Molecular Life Sciences*.

[44] Zakikhani M., Dowling R., Fantus I. G., Sonenberg N., Pollak M. (2006). Metformin is an AMP kinase–dependent growth inhibitor for breast cancer cells. *Cancer Research*.

[45] Rattan R., Giri S., Hartmann L. C., Shridhar V. (2011). Metformin attenuates ovarian cancer cell growth in an AMP-kinase dispensable manner. *Journal of Cellular and Molecular Medicine*.

[46] Sun X.-J., Zhang P., Li H. H., Jiang Z. W., Jiang C. C., Liu H. (2014). Cisplatin combined with metformin inhibits migration and invasion of human nasopharyngeal carcinoma cells by regulating E-cadherin and MMP-9. *Asian Pacific Journal of Cancer Prevention*.

[47] Trinh S. X., Nguyen H. T., Saimuang K., Prachayasittikul V., Chan On W. (2017). Metformin inhibits migration and invasion of cholangiocarcinoma cells. *Asian Pacific Journal of Cancer Prevention*.

[48] Gao L.-B., Tian S., Gao H. H., Xu Y. Y. (2013). Metformin inhibits glioma cell U251 invasion by downregulation of fibulin-3. *Neuroreport*.

[49] Khalil B. D., El-Sibai M. (2012). Rho GTPases in primary brain tumor malignancy and invasion. *Journal of Neuro-Oncology*.

[50] Hanna S., Khalil B., Nasrallah A. (2014). StarD13 is a tumor suppressor in breast cancer that regulates cell motility and invasion. *International Journal of Oncology*.

[51] Cerezo M., Tichet M., Abbe P. (2013). Metformin blocks melanoma invasion and metastasis development in AMPK/p53-dependent manner. *Molecular Cancer Therapeutics*.

[52] Wu B., Li S., Sheng L. (2012). Metformin inhibits the development and metastasis of ovarian cancer. *Oncology Reports*.

